# Associated factors, incidence, and management of gestational and congenital syphilis in a Brazilian state capital: a cross-sectional study

**DOI:** 10.1590/S1678-9946202466021

**Published:** 2024-04-19

**Authors:** Cássia de Paula Pires, Lisany Krug Mareto, Márcio José de Medeiros, Everton Falcão de Oliveira

**Affiliations:** 1 Universidade Federal de Mato Grosso do Sul, Faculdade de Medicina, Programa de Pós-Graduação Stricto Sensu em Doenças Infecciosas e Parasitárias, Campo Grande, Mato Grosso do Sul, Brazil; 2 Universidade Federal do Rio de Janeiro, Campus Macaé, Rio de Janeiro, Rio de Janeiro, Brazil; 3 Universidade Federal de Mato Grosso do Sul, Faculdade de Medicina, Campo Grande, Mato Grosso do Sul, Brazil

**Keywords:** Gestational syphilis, Congenital syphilis, Vertical transmission, Prenatal care

## Abstract

Maternal and child health remains an enduring global challenge, having occupied a prominent position on international agendas since the dawn of the 21st century. During pregnancy, syphilis emerges as the second most prevalent cause of stillbirth on a global scale, potentially leading to a range of adverse outcomes. This study aimed to describe the clinical and epidemiological profile of cases of gestational and congenital syphilis and the hospital care provided for newborns in Campo Grande municipality, Mato Grosso do Sul State, Brazil, from 2013 to 2018. This is a cross-sectional study based on data from Brazilian Notifiable Diseases Surveillance System (SINAN) and hospital medical records. Chi-square or Fisher’s exact test and logistic regression analysis were used to assess the associations and relationships between the child’s clinical outcome at birth and the mother’s clinical-obstetric and epidemiological characteristics. Cumulative detection rate of gestational syphilis was 174.3 cases per 1,000 live births and cumulative incidence of congenital syphilis was 47.7 cases per 1,000 live births. Alcoholism, prenatal care, number of prenatal visits, maternal treatment regimen, and timing of maternal diagnosis were associated with child’s clinical outcome at birth and considered in the regression model. Prenatal visits showed a protective effect against the signs and symptoms of congenital syphilis (odds ratio = 0.37; 95% confidence interval = 0.17−0.77). Medical assistance was considered inadequate in 62.3% of cases. Prenatal consultations should be encouraged among pregnant women. There is a need for better education of health personnel on the treatment and diagnosis of syphilis.

## INTRODUCTION

*Treponema pallidum* infection has been a threat to humans for many centuries^1^ and has reemerged worldwide with a continuous increase in its incidence rates since the early 2000s^2-4^. Syphilis in pregnancy is the second most common cause of stillbirth globally and also results in miscarriage, preterm delivery, low birth weight, neonatal death, and infections in newborn infants (congenital syphilis)^5,6^.

Globally, it is estimated that gestational syphilis affects more than 1 million pregnancies, with approximately 300,000 fetal and neonatal deaths per year^5^. In 2021, 74,095 cases of gestational syphilis (detection rate of 27.0/1,000 live births), 27,019 cases of congenital syphilis (incidence rate of 9.9/1,000 live births), and 192 deaths due to congenital syphilis (mortality rate of 7.0/100,000 live births) were reported in Brazil^7^. In the same year in Campo Grande municipality, one infant death and three fetal deaths related to congenital syphilis were reported, which represents a specific mortality rate of 7.8 deaths per 100,000 live births and a fetal mortality rate of 23.5 fetal deaths per 100,000 live births, respectively^7^.

Inadequate prenatal care is one of the major factors for the high incidence rate of congenital syphilis^8^. Factors such as low socioeconomic status, drug abuse, and irregular health check-ups are also associated with congenital syphilis^8-10^. In the absence of treatment, the risk for vertical transmission of *T. pallidum* is high; however, timely diagnosis and adequate treatment can reduce the probability of vertical transmission^6^.

Maternal and child health remains an enduring global challenge, having occupied a prominent position on international agendas since the dawn of the 21^st^ century^11^. Exploratory studies focusing on intrauterine and neonatal adverse outcomes^11^, relying on retrospective data sources such as hospital records, provide valuable means of identifying deficiencies or breakdowns in the healthcare system. These analyses highlight critical areas where interventions are essential for enhancing maternal and child health outcomes. Therefore, this study aimed to describe the clinical and epidemiological profiles of gestational and congenital syphilis cases, as well as to outline the management and hospital care for these infants in Campo Grande municipality, a Brazilian capital city situated in the Brazil-Paraguay-Bolivia border area.

## MATERIALS AND METHODS

### Study design and population

This is a cross-sectional study based on data from Brazilian Notifiable Diseases Surveillance System (SINAN) and the medical records of the congenital and gestational syphilis cases from Campo Grande municipality, Mato Grosso do Sul State, Brazil, from 2013 to 2018. Data from medical records were obtained from the four main maternity hospitals (two exclusively public and the other two public and private) in Campo Grande municipality, which collectively account for approximately 95% of total deliveries performed in the city^12^. In Brazil, prenatal care, including syphilis testing in the first, second, and third trimesters of pregnancy and treatment of positive cases, is provided entirely free of charge by Brazilian Unified Health System (SUS).

The clinical case definition employed were those in force from 2013 to 2018^13,14^. In conjunction with the laboratory confirmation documented in the medical record and/or notification form, the identification of at least one sign or symptom consistent with congenital syphilis was used as the symptomatic case definition for congenital syphilis. These included reports on the congenital syphilis notification form or laboratory findings and clinical investigations suggestive of anemia, jaundice, splenomegaly, osteochondritis, rhinitis with bloody-mucus discharge, hepatomegaly, skin lesions, pseudo paralysis, radiographic abnormalities of long bones, and alteration of cerebrospinal fluid.

For this study, the criteria for the definition of inadequate treatment of pregnant women was the same used by Brazilian Ministry of Health at the time of the data collection period: (1) treatment with any drug other than penicillin; (2) incomplete treatment, even with penicillin; (3) inadequate treatment for the clinical stage of the disease; (4) treatment onset within 30 days prior to delivery; and (5) sexual partner(s) with untreated or inadequately treated syphilis^13,14^. Cases with incomplete notification forms in SINAN were excluded.

### Study data

Data were extracted from SINAN and from the hospital medical records, including: maternal socioeconomic and demographic data, clinical obstetric and infant records, and method of diagnosis and treatment of both mothers and infants.

Data from the Brazilian Live Births Information System (SINASC) on the total number of live births during the study period was used to calculate the disease frequency measures used in this study. Manual record linkage was conducted to integrate the data from SINAN and medical records. When inconsistencies were found, data from the medical record was considered since newborn’s hospital records are filled out based on mothers’ prenatal booklet. This procedure reduced possible filling errors and increased the quality of the data used in the study.

### Data analysis

Frequency distribution tables were used to describe the analyzed variables and to characterize the study population. Detection and incidence rates were used as measures of the frequency of gestational and congenital syphilis, respectively. The annual detection rate of syphilis in pregnant women was calculated by dividing the number of syphilis cases in women reported each year in SINAN database by the total number of live births in that year. For the crude annual incidence rate of congenital syphilis, the number of congenital syphilis cases reported in SINAN database was divided by the total number of live births in that year.

Cumulative frequency measures were also estimated, with the sum of confirmed cases in the study period as the numerator and the number of live births during the study period (which, in this case, corresponds to the average number of live births for 2015 and 2016) as the denominator.

The chi-square test was used to assess the statistical association between the variables being studied. Fisher’s exact test was used when assumptions for chi-square test were not met. The odds ratio (OR) was also used as an association measure.

The binomial logistic regression model was used to assess the association between the child’s clinical outcome at birth (dependent variable) and the mother’s data (independent variables). Initially, the variables with p-value ≤ 0.20 in the association analysis were included in the modeling stage. The stepwise algorithm (considering both backward and forward directions) was adopted, using the Akaike’s information criterion (AIC), as an initial procedure for the selection of the explanatory variables for the model. The final model was the one with the lowest AIC value. As a measure of goodness of fit, the Hosmer-Lemeshow test was adopted. A 5% significance level (α = 0.05) along with 95% confidence intervals were adopted for all hypothesis tests. The analysis was performed using R programming language (version 4.0.4, R Core Team, Vienna, Austria) and the *descr* and *generalhoslem* packages were used.

The medical assistance related to the adequacy of hospital management and conduct was verified by analyzing case by case to check if all the steps described in the Brazilian guidelines for the management of maternal and congenital syphilis were met. The proportion of adequacy of hospital conduct was estimated as the ratio between the number of infants who received all procedures recommended by Brazilian Ministry of Health and the total number of infants who had their medical records reviewed.

### Ethics approval and consent to participate

The study was approved by the Research Ethics Committee of the Federal University of Mato Grosso do Sul, Brazil, and procedures were in accordance with the ethical standards of this committee and with the Helsinki Declaration of 1975, as revised in 1983. Informed consent has been waived by the Research Ethics Committee of the Federal University of Mato Grosso do Sul.

## RESULTS

From 2013 to 2018, 2,458 cases of gestational syphilis and 672 cases of congenital syphilis were reported to SINAN. Of the 672 cases of congenital syphilis, 346 (51.5%) medical records were identified for review. However, only 324 were included in the study as 22 medical records were excluded due to data unavailability.

**Figure 1:**
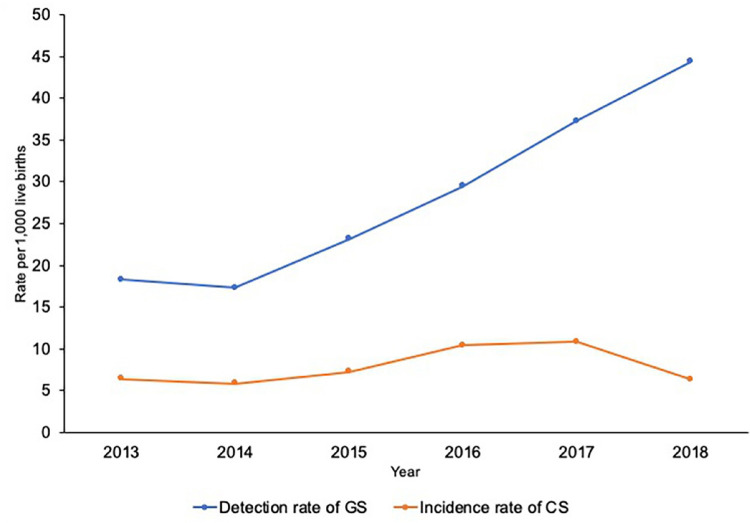
Detection rate of gestational syphilis and incidence rate of congenital syphilis per 1,000 live births.

During the study period, a marked and continuous increase in the detection rate of gestational syphilis was observed (except in 2014), especially from 2016 onward. The cumulative detection rate for the study period was 174.3/1,000 live births. The incidence rate of congenital syphilis increased more discreetly over the study period, marked by declines from 2013 to 2014 and again from 2017 to 2018 (Figure 1 and Supplementary Table S1). The cumulative incidence rate for the study period was 47.7/1,000 live births.

### Gestational syphilis

Table 1 shows the sociodemographic data of pregnant women with syphilis. The age group of 20 to 29 years had the highest frequency of cases (52.1%; mean = 24.1 years; median = 23 years; standard deviation = 6.4 years). Regarding ethnicity/skin-color, 57.8% women were Mixed-race. Nearly one-third of pregnant women (39.6%) had up to nine years of education, which included the non-literate, complete, and incomplete elementary education categories (Table 1).

**Table 1:** Epidemiological and clinical data of gestational congenital syphilis cases.

Gestational syphilis (SINAN data)	*n*	%
**Maternal Age (year)**		
10 to 19	673	27.4
20 to 29	1281	52.1
30 to 39	450	18.3
40 and more	54	2.2
**Ethnicity/Skin Color**		
White	714	29.0
Black	159	6.5
Mixed-race	1,420	57.8
Yellow	33	1.3
Indigenous	29	1.2
Not informed	103	4.2
**Education (years)**		
Up to 9	972	39.6
10 to 12	895	36.4
13 and more	86	3.5
Not informed	505	20.5
**Trimester of diagnosis of treponemal infection**		
First	968	39.4
Second	695	28.3
Third	634	25.8
Not informed	161	6.5
**Clinical phase of syphilis**		
Primary	328	13.3
Secondary	53	2.2
Tertiary	669	27.2
Latent	947	38.5
Not informed	461	18.8
**Treponemal test**		
Positive	2,298	93.5
Non-reactive	28	1.1
Not informed	132	5.4
**Non-treponemal test**		
Positive	1,329	54.1
Non-reactive	235	9.5
Not informed	894	36.4
**Adequacy of treatment**		
Adequate	1,496	60.9
Inadequate	962	39.1
Total	2,458	-
**Congenital syphilis (medical record data)**		
**Sex**		
Female	161	49.7
Male	156	48.1
Not informed	7	2.2
**Ethnicity/skin color**		
White	98	30.3
Black	4	1.2
Mixed-race	187	57.7
Indigenous	1	0.3
Not informed	34	10.5
**Birth weight**		
SGA < 2500g	62	19.1
AGA	230	71.0
LIG > 4000g	7	2.2
Not informed	4	1.2
Not applicable (miscarriage/stillbirth)	21	6.5
**Gestational age at birth**		
Pre-term	66	20.4
Full-term	229	70.7
Post-term	8	2.5
Not informed (miscarriage/stillbirth)	21	6.5
**Apgar score**		
Adequate	166	51.2
Not suitable	14	4.3
Not informed	144	44.4
Total	324	-

SGA = small for gestational age; AGA = appropriate for gestational age; LIG = large for gestational age; APGAR = appearance (skin color), pulse (heart rate), grimace response (reflexes), activity (muscle tone), respiration (breathing rate and effort).

Among the cases reported in SINAN, most pregnant women (54.1%) were in the second and third trimesters of pregnancy when syphilis was diagnosed. Regarding the clinical stage, syphilis was classified as latent and tertiary in 38.5% and 7.2% of the cases, respectively.

The treponemal tests showed a positive result in 93.5% of the cases, whereas the non-treponemal tests were positive in 54.1% of the cases. Only 1,496 pregnant women (60.9%) were adequately treated (Table 1).

The syphilis notification form for pregnant women does not contain specific fields to verify the completion of prenatal care and the number of consultations performed until the date of diagnosis. A field is available to indicate the code of the health unit where prenatal care was provided. This code data showed that approximately 2,185 pregnant women (88.9%) had at least one prenatal consultation. Thus, it is not possible to identify the performance and adequacy of the number of prenatal consultations from SINAN. Therefore, data on the number of prenatal consultations were evaluated only based on the review of the 324 hospital medical records: most pregnant women had prenatal consultations (80.2%). However, 36.1% had less than six consultations, and 28.4% of the women had six or more consultations.

The maternal profile pertaining to prior complications and substance use (alcohol, tobacco, and drugs) was also derived from data extracted from the analysis of 324 medical records. Most pregnant women had a history of syphilis during previous pregnancies or had newly acquired syphilis (67.6%). Other complications, such as urinary tract infection, STORCH (syphilis, toxoplasmosis, other infections, rubella, cytomegalovirus infection, and herpes simplex), and pregnancy-specific hypertensive disease were reported less frequently (15.7%, 13.5%, and 8.3%, respectively).

More than half (68.2%) participants denied using any type of substance; however, a considerable number of pregnant women reported using at least one chemical substance during pregnancy (23.1%) and 7.9% reported the combined use of two or more substances. The substances mentioned included cigarettes (15.1%), alcohol (6.7%), coca derivatives (5.2%), and marijuana (2.1%).

Supplementary Table S2 presents the results of the hypothesis tests conducted to verify and identify possible factors associated with the outcome of the maternal treatment regimen. Initially, there was a significant association (p-value < 0.001) between the treatment regimen and time of diagnosis, possibly due to the high number of pregnant women who were diagnosed only at the time of delivery or during the dilation and curettage procedure, after the delivery. Therefore, these mothers did not receive drug treatment during pregnancy. It is also worth noting that some pregnant women were diagnosed during prenatal care and were still inadequately treated. The other variables significantly associated with the treatment regimen were the completion of prenatal care and number of prenatal consultations. With these variables, it is possible to descriptively observe the relationship between the inadequacy or non-performance of the treatment and the low number of prenatal consultations.

### Congenital syphilis

Among the 342 medical records reviewed, the proportion of female infants (49.7%) was not much different from the male infants (48.1%). Regarding ethnicity/skin-color, most were Mixed-race. Moreover, most infants were born at the appropriate gestational age with adequate birth weight and Apgar score, which measures appearance, pulse, grimace, activity, and respiration (Table 1).

Table 2 presents additional clinical and laboratory data from both SINAN and the medical records of confirmed congenital syphilis. Prenatal care was performed in 75.4% (*n* = 507) of the reported cases. As there is no field for the number of consultations in the SINAN notification form, it was not possible to verify whether the prenatal care occurred as recommended, with a minimum of six consultations.

**Table 2:** Clinical and laboratory data of confirmed congenital syphilis cases (SINAN: *n* = 672; hospital medical records: *n* = 324).

Parameter	Source	

	SINAN *n* (%)	Medical records *n* (%)
**Notification location**		
Tertiary care	623 (92.7)	-
Primary care	49 (7.3)	-
**Maternal diagnosis**		
During prenatal	383 (57.0)	-
At the time of delivery/curettage	225 (33.5)	-
After delivery	54 (8.0)	-
Not informed	10 (1.5)	-
**Non-treponemal test (blood)**		
Positive	453 (67.4)	250 (77.1)
Non-reactive	121 (18.0)	45 (13.8)
Not informed	98 (14.6)	2 (0.6)
Not performed	-	27 (8.3)
**Non-treponemal test (liquor)**		
Reagent	29 (4.3)	18 (5.4)
Non-reactive	325 (48.4)	213 (65.7)
Not informed	318 (47.3)	22 (6.8)
Not performed	-	71 (21.9)
**Non-treponemal (blood) test titration**		
1/8 or more	-	108 (33.3)
Less than 1/8	-	142 (43.8)
Non-reactive	-	45 (13.8)
Not performed	-	27 (8.3)
Not informed	-	2 (0.6)
**Non-treponemal (liquor) test titration**		
1/8 or more	-	10 (3.0)
Less than 1/8	-	8 (2.4)
Non-reactive	-	213 (65.7)
Not performed	-	71 (21.9)
Not informed	-	22 (6.8)
**Serological titer (ascending titration)**		
Yes	-	14 (4.3)
No	-	20 (6.1)
Not performed	-	269 (83.0)
Not applicable	-	21 (6.4)
**Microscopy detection of *T. pallidum***		
Yes	-	3 (0.9)
No	-	88 (27.1)
Not performed	-	209 (64.5)
Not applicable	-	21 (6.4)
**Clinical classification**		
Symptomatic	98 (14.6)	-
Asymptomatic	497 (73.9)	-
Not applicable* or not informed	77 (11.5)	-

This table includes cases of miscarriage and stillbirth since, in some cases (depending on gestational age), umbilical cord blood was collected. Only ascending titration and microscopy detection of *T. pallidum* were not performed in these cases; *Includes stillbirth and miscarriage.

In agreement with the results presented for gestational syphilis, the data obtained from SINAN for congenital syphilis indicated that gestational syphilis was diagnosed during prenatal care in approximately half of the cases, which may suggest failures in prenatal care since the remainder (more than 40%) were diagnosed at or after delivery (Table 2). Maternal treatment during pregnancy was not performed or was performed inappropriately in 39.4% (*n* = 265) and 58.5% (*n* = 395) of the cases, respectively, which indicates poor therapeutic management in 97.9% of the cases.

Non-treponemal reactive tests, performed using the infant’s blood, were frequently reported in both data sources (Table 2). The test titration was ≥ 8 in 33.3% (*n* = 108) of congenital syphilis cases. The non-treponemal tests using liquor were most frequently non-reactive. Ascending titration was not performed in more than 80% of the cases, while an increase in the titer was noted in 4.3% of the infants. Dark field microscopy was not used to demonstrate *T. pallidum* in 64.5% of the cases, with a non-reactive result obtained in 27.1% (Table 2).

Considering the data from SINAN, most (74%) newborns were classified as asymptomatic after birth (Table 2). A total of 74 negative outcomes were found, including miscarriage (*n* = 30), stillbirth (*n* = 39), and mortality from congenital syphilis (*n* = 5). To complement these findings, data from medical records were analyzed to identify at least one symptom that could be associated with congenital syphilis. Based on medical records review, 50.9% were classified as asymptomatic for congenital syphilis. Among infants who were symptomatic at birth (42.6%), jaundice was the most frequent symptom, followed by skin lesions and anemia (Table 3).

**Table 3:** Symptomatology presented by cases of congenital syphilis based on the review of medical records ( = 324).

Signs and symptoms	Presence of signs and symptoms			

	Yes		No	

	*n*	%	*n*	%
Jaundice	111	34.3	192	59.3
Anemia	28	8.7	275	84.9
Splenomegaly	4	1.3	299	92.3
Osteochondritis	1	0.4	302	93.2
Muco-bloody rhinitis	1	0.4	302	93.2
Hepatomegaly	8	2.5	295	91.1
Skin lesions	32	9.9	271	83.7
Pseudoparalysis	-	-	303	93.6

Supplementary Table S3 presents the results of the associations between the clinical outcome of the disease (symptomatic or asymptomatic) and the studied covariates. Among the assessed covariates, alcohol consumption, prenatal care, number of prenatal consultations, and maternal treatment regimen showed a significant association with the clinical outcome of the disease. Considering the OR and its confidence intervals, only protective factors were associated with the outcome.

As indicated in the Material and Methods section, the variables that presented p-value ≤ 0.20 were included in the binomial logistic regression model used to assess the association between the infant’s clinical outcome at birth and the mother’s data (Supplementary Table S3). Therefore, in addition to the covariates previously described, the following were included: substance use (any substances, including alcohol and tobacco products) and timing of maternal diagnosis. However, only the number of prenatal consultations remained in the final model as an independent variable (Table 4), showing the best fit among all the combinations performed (AIC = 322.7).

**Table 4:** Final model for the child’s clinical outcome at birth.

Parameter	Regression coefficient		OR (95% CI)

	β	*p*-value	
Prenatal consultations (6 or more)	−1.050	< 0.001	0.37 (0.17–0.77)
Prenatal consultations (less than 6)	−0.787	< 0.05	0.49 (0.23–0.98)
Prenatal consultations (not performed)	0.693	< 0.05	Reference

The logistic regression model results reinforce the possible protective action of prenatal consultations against the occurrence of signs and symptoms related to congenital syphilis (Table 4). As the reference category of the outcome (dependent variable) for the construction of the model was the symptomatic category, the negative sign of the β coefficients for the categories including ≥ 6 and < 6 consultations show that higher number of prenatal consultations present lower odds of symptomatic live births. By analyzing the OR, the measure with the greatest magnitude (that is, furthest from 1 and closest to 0) and, therefore, with the greatest potential protective effect is that of the category of ≥ 6 consultations. According to the goodness of fit by Hosmer-Lemeshow test, the p-value (0.639) was not significant, indicating that the logistic regression model was well adjusted.

Regarding the possible success rate in blocking vertical transmission with the therapeutic protocol recommended by Brazilian Ministry of Health, only five (7.9%) of 63 adequately treated pregnant women (who constitute 19.4% of all pregnant women with gestational syphilis) had infants who were asymptomatic at birth and had non-reactive results in the treponemal and non-treponemal tests.

In the treatment of infants, the most frequently administered regimen was crystalline penicillin (100,000 to 150,000 IU for 10 days), which was adopted in 248 cases (76.5%). This was followed by benzathine penicillin (50,000 IU in a single dose) in 26 cases (8.0%) and procaine penicillin (50,000 IU for 10 days) in six infants (1.8%). Additionally, an alternative regimen was reported in 16 cases (4.9%), while data pertaining to the pharmacological treatment of infants was not provided in 8.6% of the cases (28).

Concerning the protocol for the management of congenital syphilis, medical assistance (hospital care) was classified as inadequate in 62.3% (*n* = 202) of cases and as adequate in 32.4% (*n* = 105) of cases, while miscarriages and stillbirths, accounting for 5.2% (*n* = 17) of cases, were categorized as not applicable. The inadequacy primarily stemmed from the omission of essential neonatal screening tests, including long bone radiography, lumbar puncture, and hemogram, in addition to misuse of penicillin therapy. Radiographic analysis of long bones was conducted in most cases (73.4%). However, in terms of screening for newborn infants, hearing tests were performed less frequently (5.0%), and eye tests were even less commonly performed (2.8%).

## DISCUSSION

Our findings reinforce the importance of strategies that are already strongly recognized as important for the maintenance of good maternal and child health. Several factors, both known and potentially unknown, may have contributed to the increase in syphilis cases worldwide. This includes, but is not limited to, the global penicillin shortage that occurred in 2015–2017^15^. In Brazil, one of these factors may be related to an important change in the investigation of congenital syphilis cases in 2017: the exclusion of the treatment of the mother’s sexual partner as a case definition criteria^16^ may have adversely affected active search and follow-up for partner treatment. This change could have potentially hindered efforts in controlling syphilis, given that active partner engagement and treatment are crucial challenges in the management of syphilis in pregnant women, consequently influencing the prevention of congenital syphilis.

Easy access to healthcare services and good quality prenatal care, including during the period of delivery, is crucial for reducing the burden of congenital syphilis. The high incidence of congenital syphilis, especially related to symptomatic cases, indicates failures in the healthcare system^17,18^. High incidences of gestational and congenital syphilis with a tendency to increase over time have been observed worldwide^4^. In Brazil, from 2007 to 2017, the incidence rate of gestational syphilis had an expressive increase of 660%, from 2.2 to 16.9 per 1,000 live births^3^. However, the incidence and risk of gestational syphilis in Campo Grande municipality were higher when compared to national data. This fact emphasizes potential shortcomings in disease control, including factors associated with access to diagnosis and early treatment for pregnant women and their partners. Moreover, the situation heightens the risk of adverse outcomes related to congenital syphilis.

Despite the Brazilian guidelines for the management and control of maternal and congenital syphilis, which predicted a reduction in the incidence rate of congenital syphilis to 0.5 cases per 1,000 live births by 2015^19^, the incidence observed in this study was approximately 20 times greater than the targeted incidence, reaching 10.8 per 1,000 live births in 2017. This increase may be associated with the shortage of penicillin, despite being prioritized for use only in pregnant women and newborns with syphilis. In low-income regions, the shortage was even more severe^20^. Furthermore, the modest increase in congenital syphilis cases in Campo Grande municipality from 2014 to 2017 may reflect the initiative that prioritized penicillin use for treating pregnant women during the medication shortage period.

Demographic and socioeconomic profiles of pregnant women and children in our study are similar to other studies suggesting that pregnant women with syphilis are usually poorly educated and live in vulnerable situations, hindering their adhesion to recommended guidelines during prenatal care, as well as disease management^8,9^. Considering this profile, different strategies are necessary to reach more vulnerable populations and minimize inequalities, aiming to facilitate greater access to healthcare services^9^.

Prenatal care is essential for the control and management of *T. pallidum* infection. When performed inappropriately, it becomes one of the major factors for the increase in the incidence of congenital syphilis^5,6^. Guimarães *et al*.^21^, considered prenatal care in Brazil as inadequate, especially in relation to the access and quality offered, and highlighted the lack of adequate infrastructure in the Midwest region of Brazil. This finding should be discussed since prenatal care was the only variable maintained in our final logistic regression model, suggesting that it is a protective factor against the occurrence of symptoms of congenital syphilis in newborn infants.

Regarding the Venereal Disease Research Laboratory (VDRL) test during pregnancy, some authors have suggested that pregnant women with titers above 1/4 have a four times greater risk of adverse pregnancy outcomes than those with a non-reactive result^22^. A high titer may indicate recent infection, with a greater risk of transmission to the fetus and, consequently, a greater probability of mortality related to the inflammatory response and reduced placental blood flow^23^. Thus, the importance of early detection and adequate treatment is highlighted, including the follow-up of pregnant women with low VRDL titers (less than 1/8).

The association of substance use during pregnancy and increased risk of gestational and congenital syphilis have been reported in previous studies^24^. Some studies have indicated that drug use is associated with non-compliance of prenatal consultations, which is a risk factor for adverse pregnancy outcomes, demonstrating the vulnerability of these women and the need for public policies specific to this population^5,8,25^.

Congenital syphilis is an important cause of premature birth and low birth rate since it is associated with higher frequencies of perinatal deaths^26^. In our study, 20.3% of the infants were born with a gestational age of < 37 weeks, and almost 20% had low birth weight (< 2,500 g), a value higher than that found in other studies^27,28^. Among infants classified as symptomatic, our findings are similar to other studies that reported jaundice as the most frequent outcome, followed by anemia and problems in the bone structure^18,29^.

Early and adequate screening for syphilis during pregnancy proved to be highly cost-effective, with costs of less than USD 60, including screening and full treatment^30^. Thus, the importance of prenatal care for the prevention, treatment, and follow-up of syphilis is once again reiterated, which is one of the best (if not the best) control strategies currently available.

Regarding the clinical management of cases of congenital syphilis as recommended by the Brazilian Ministry of Health, it was observed that most cases were not adequately managed in the hospital environment, especially due to the failure to perform neonatal screening tests. Additionally, the drug treatment protocol was considered inadequate in most cases, which suggests a need for the administration of sufficient treatment following the criteria recommended by Brazilian health authorities.

This study presents a few limitations, as is common with research conducted using secondary data. Some gestational and congenital syphilis notification forms in the SINAN database were not fully completed. Moreover, a lack of clarity and organization was found in the medical records, which highlights the need for improved accuracy in these records as it should be the best source of existing information. The use of medical records can be a way to minimize the SINAN data loss and improve the understanding of the profiles of pregnant women with syphilis. The descriptive nature of our study design precludes us from making inferences, such as identifying risk or protective factors. In addition, data on miscarriages and stillbirths had to be excluded from the univariate analysis and modeling, as it was not possible to state the cause of these events, even if congenital syphilis caused the miscarriages and prematurity. This exclusion, coupled with underreporting cases, may have led to an underestimation of the measures of association and other analyses conducted in the study.

## CONCLUSION

The findings of our study can be crucial for shaping public health strategies to control syphilis, especially in promoting and increasing the coverage of prenatal care, which can indirectly contribute to the diagnosis and treatment of the sexual partners of pregnant women diagnosed with syphilis. Our study may be useful for generating hypotheses for later, as well as more rigorous and prospective studies, particularly concerning the effect of the number of prenatal appointments on the prevention of congenital syphilis. Despite the increased coverage of prenatal care in Brasil^10^, the recommended number of appointments is not consistently adhered to during pregnancy. In addition, further studies on strategies for the control, follow-up, and treatment of syphilis are needed in different study areas, such as clinical-epidemiological investigations into the occurrence of other clinical forms of syphilis (primary, secondary, and tertiary).
