# Supplementary material for: Associated factors, incidence, and management of gestational and congenital syphilis in a Brazilian state capital: a cross-sectional study
Source: Rev Inst Med Trop Sao Paulo. 2024 Apr 19;66:e21. doi: 10.1590/S1678-9946202466021 (PMC11027491; doi:10.1590/S1678-9946202466021)
Supplement: Supplementary file 1 [file 1678-9946-rimtsp-66-S1678-9946202466021-supl-mat.pdf]

REVISTA DO INSTITUTO DE MEDICINA TROPICAL DE SÃO PAULO

JOURNAL OF THE SÃO PAULO INSTITUTE OF TROPICAL MEDICINE

SUPPLEMENTARY MATERIAL

https://doi.org/10.48331/scielodata.WBIXAD

Associated factors, incidence, and management of gestational and congenital syphilis in a Brazilian state capital: a cross-sectional study

Cássia de Paula Pires [1], **Lisany Krug Mareto**[1], **Márcio José de Medeiros** [2], **Everton Falcão de Oliveira** [1,3]

[1]Universidade Federal de Mato Grosso do Sul, Faculdade de Medicina, Programa de Pós-Graduação Stricto Sensu em Doenças Infecciosas e Parasitárias, Campo Grande, Mato Grosso do Sul, Brazil

[2]Universidade Federal do Rio de Janeiro, Campus Macaé, Rio de Janeiro, Rio de Janeiro, Brazil

[3]Universidade Federal de Mato Grosso do Sul, Faculdade de Medicina, Campo Grande, Mato Grosso do Sul, Brazil

Correspondence to: Everton Falcão de Oliveira
Universidade Federal de Mato Grosso do Sul, Faculdade de Medicina, Av. Costa e Silva, Cidade Universitária, CEP 79070-900, Campo Grande, MS, Brazil

E-mail: everton.falcao@ufms.br

Received: 25 September 2023

Accepted: 22 January 2024

Supplementary Table S1 - Frequency of gestational and congenital syphilis cases, live births, detection rate of gestational syphilis, and incidence rate of congenital syphilis per 1,000 live births, according to year of notification.

Year	Confirmed cases of GS (%)	Confirmed cases of CS (%)	Live births	Detection rate of GS	Incidence rate of CS
2013	251 (10.2)	88 (13.1)	13,693	18.3	6.4
2014	246 (10.0)	83 (12.4)	14,203	17.3	5.8
2015	335 (13.6)	105 (15.6)	14,470	23.1	7.2
2016	405 (16.5)	143 (21.3)	13,728	29.5	10.4
2017	533 (21.7)	155 (23.1)	14,264	37.3	10.8
2018	688 (28.0)	98 (14.6)	15,473	44.4	6.3
Accumulated from the period	2,458 (100)	672 (100)	-	174.3	47.7

GS = gestational syphilis; CS = congenital syphilis.

This is an open-access article distributed under the terms of the Creative Commons Attribution License.

Supplementary Table S2 - Maternal treatment according to clinical and obstetric variables of pregnant women with syphilis (data from hospital medical records; n = 324).

	Maternal treatment			
	**Adequately treated n (%)**	**Inadequately treated n (%)**	**Untreated n (%)**	***p*-value**
**Time of maternal diagnosis**				
During prenatal	62 (31.5)	105 (53.3)	31 (15.2)	<0.001[a]
At the time of delivery/curettage	1 (0.9)	20 (18.7)	87 (80.4)	
After delivery/curettage	0	1(6.2)	15 (93.8)	
Not performed	0	1(100.0)	0	
**Non-treponemal test titration**				
Non-reactive	8 (26.7)	11 (36.7)	11 (36.7)	0.111[a]
Less than 1/8	27 (27.3)	37 (37.4)	35 (35.4)	
1/8 or more	26 (14.7)	72 (40.7)	79 (44.6)	
Not performed	2 (18.2)	5 (45.5)	4 (36.4)	
**Prenatal care**				
No	0	8 (14.3)	48 (85.7)	<0.001[b]
Yes	62 (24.0)	117 (45.3)	79 (30.6)	
Unindicated	1 (14.3)	2 (28.6)	4 (57.1)	
**Prenatal consultations**				
6 or more	32 (35.6)	36 (40.0)	22 (24.4)	<0.001[b]
Less than 6	21 (17.9)	57 (48.7)	39 (33.3)	
Not performed	0	8 (14.3)	48 (85.7)	
Not informed	10 (17.2)	26 (44.8)	22 (37.9)	
**Substance use**				
No	46 (20.9)	92 (41.8)	82 (37.3)	0.104[b]
Yes	9 (12.0)	29 (38.7)	37 (49.3)	
**Education (years)**				
0	0	1 (50.0)	1 (50.0)	0.294[a]
1 to 9	17 (14.9)	41 (36.0)	56 (49.1)	
10 to 12	15 (16.9)	41 (46.1)	33 (37.1)	
13 and more	2 (50.0)	1 (25.0)	1 (25.0)	

[a]Fisher's exact test; [b]Chi-square test.

Supplementary Table S3 - Clinical outcome of the child at birth according to covariates evaluated in the study (n = 303*).

	Clinical outcome of the child at birth		OR (95% CI)	*p*-value
	Asymptomatic n (%)	Symptomatic n (%)		
**Education**				
0	1 (50.0)	1 (50.0)	REF	0.272
1 to 9	54 (51.4)	51 (48.6)	0.94 (0.04 24.31)	
10 and 12	47 (54.7)	39 (45.3)	0.83 (0.03 – 21.44)	
13 and more	4 (100.0)		0.00 (0.00 – 12501.62)	
**Previous complications**				
No	95 (57.6)	70 (42.4)	REF	0.248
Yes	70 (50.7)	68 (49.3)	1.32 (0.84 – 2.08)	
**Pregnancy-specific hypertensive disease**				
No	152 (54.7)	126 (45.3)	REF	0.836
Yes	13 (52.0)	12 (48.0)	1.11 (0.48 – 2.54)	
**Urinary tract infection**				
No	140 (55.6)	112 (44.4)	REF	0.442
Yes	25 (49.0)	26 (51.0)	1.30 (0.71 – 2.38)	
**STORCH**				
No	144 (55.0)	118 (45.0)	REF	0.737
Yes	21 (51.2)	20 (48.8)	1.16 (0.60 – 2.25)	
**Sexually transmitted infections**				
No	162 (54.4)	136 (45.6)	REF	0.999
Yes	3 (60.0)	2 (40.0)	0.79 (0.10 – 4.86)	
**Mental disorders**				
No	163 (55.1)	133 (44.9)	REF	0.252
Yes	2 (28.6)	5 (71.4)	3.06 (0.65 – 21.63)	
**Syphilis reinfection**				
No	113 (54.1)	96 (45.9)	REF	0.740
Yes	22 (51.2)	21 (48.8)	1.12 (0.58 – 2.17)	
**Substance use**				
No	117 (56.2)	91 (43.8)	REF	0.137
Yes	34 (45.9)	40 (54.1)	1.51 (0.89 – 2.59)	
**Alcoholism**				
No	144 (55.4)	116 (44.6)	REF	0.044
Yes	7 (31.8)	15 (68.2)	2.66 (0.89 – 2.59)	
**Smoking**				
No	126 (53.8)	108 (46.2)	REF	0.874
Yes	25 (52.1)	23 (47.9)	1.07 (0.57 – 2.00)	
**Cocaine derivatives use**				
No	142 (53.6)	123 (46.4)	REF	0.999
Yes	9 (52.9)	8 (47.1)	1.03 (0.37 – 2.76)	
**Marijuana use**				
No	147 (53.5)	128 (46.5)	REF	0.999
Yes	4 (57.1)	3 (42.9)	0.86 (0.17 – 3.96)	

Supplementary Table S3 - Clinical outcome of the child at birth according to covariates evaluated in the study (n = 303*). (cont.)

	Clinical outcome of the child at birth		OR (95% CI)	*p*-value
	Asymptomatic n (%)	Symptomatic n (%)		
**Psychotropics use**				
No	151 (53.7)	130 (46.3)	--	0.465
Yes	0 (0.0)	1 (100.0)	--	
**Partner treatment for syphilis**				
No	104 (53.1)	92 (46.9)	REF	0.999
Yes	46 (52.3)	42 (47.7)	1.03 (0.62 – 1.71)	
**Ethnicity/skin color (infants)**				
White	50 (53.2)	44 (46.8)	--	0.769
Mixed-race	99 (56.6)	76 (43.4)	--	
Black	3 (75.0)	1 (25.0)	--	
Indigenous	1 (100.0)	0 (0.0)	--	
**Prenatal care**				
No	16 (35.6)	29 (64.4)	REF	0.009
Yes	143 (57.2)	107 (42.8)	0.41 (0.21 – 0.79)	
**Prenatal consultations**				
6 and more	55 (59.8)	37 (40.2)	0.37 (0.17 – 0.77)	0.029
Less than 6	60 (53.1)	53 (46.9)	0.49 (0.23 – 0.98)	
Not performed	16 (35.6)	29 (64.4)	REF	
**Time of maternal diagnosis**				
During prenatal	112 (58.9)	78 (41.1)	--	0.076
At the time of delivery/curettage	45 (45.5)	54 (54.5)	--	
After delivery/curettage	7 (58.3)	5 (41.7)	--	
Not performed	0 (0.0)	1 (100.0)	--	
**Non-treponemal test titration (pregnant women)**				
1/8 and more	85 (52.5)	77 (47.5)	REF	0.673
Less than 1/8	56 (56.0)	44 (44.0)	0.87 (0.52 – 1.42)	
Non-reactive	18 (64.3)	10 (35.7)	0.61 (0.26 – 1.39)	
Not performed	4 (50.0)	4 (50.0)	1.10 (0.25 – 4.81)	
**Non-treponemal test at delivery (pregnant women)**				
Positive	143 (54.0)	122 (46.0)	1.54 (0.70 – 3.58)	0.591
Non-reactive	18 (64.3)	10 (35.7)	REF	
Not performed	4 (50.0)	4 (50.0)	1.80 (0.36 – 9.21)	
**Treponemal test at delivery (pregnant women)**				
Positive	83 (55.0)	68 (45.0)	2.05 (0.43 – 14.61)	0.757
Non-reactive	5 (71.4)	2 (28.6)	REF	
Not performed	73 (55.3)	59 (44.7)	2.02 (0.42 – 14.46)	
**Maternal treatment**				
Adequately treated	36 (57.1)	27 (42.9)	REF	0.028
Inadequately treated	76 (61.8)	47 (38.2)	0.82 (0.44 – 1.53)	
Not performed	51 (44.7)	63 (55.3)	1.65 (0.89 – 3.08)	

Supplementary Table S3 - Clinical outcome of the child at birth according to covariates evaluated in the study (n = 303*). (cont.)

	Clinical outcome of the child at birth		OR (95% CI)	*p*-value
	Asymptomatic n (%)	Symptomatic n (%)		
**Non-treponemal test at delivery (infants)**				
Positive	134 (53.6)	116 (46.4)	1.08 (0.57 – 2.07)	0.385
Non-reactive	25 (55.6)	20 (44.4)	REF	
Not performed	5 (83.3)	1 (16.7)	0.25 (0.01 – 1.72)	
**Non-treponemal test titration at delivery (infants)**				
1/8 and more	53 (49.1)	55 (50.9)	REF	0.318
Less than 1/8	81 (57.0)	61 (43.0)	0.73 (0.44 – 1.20)	
Non-reactive	25 (55.6)	20 (44.4)	0.77 (0.38 – 1.55)	
Not performed	5 (83.3)	1 (16.7)	0.19 (0.01 – 1.25)	
**Non-treponemal test using liquor at delivery (infants)**				
Positive	12 (63.2)	7 (36.8)	0.70 (0.25 – 1.80)	0.706
Non-reactive	116 (54.5)	97 (45.5)	REF	
Not performed	37 (52.1)	34 (47.9)	1.10 (0.64 – 1.88)	
**Non-treponemal test titration using liquor at delivery (infants)**				
1/8 and more	8 (80.0)	2 (20.0)	REF	0.307
Less than 1/8	3 (37.5)	5 (62.5)	6.67 (0.91 – 69.73)	
Non-reactive	116 (54.5)	97 (45.5)	3.34 (0.81 – 22.51)	
Not performed	37 (52.1)	34 (47.9)	3.68 (0.85 – 25.51)	

*Stillbirths and miscarriage were removed from this stage of the analysis; OR = odds ratio; 95% CI = 95% confidence interval for OR; REF = reference category for the OR calculation; -- = it was not possible to calculate the OR with the data of the observed sample.
